# From Theory to Practice: An Implementing Case-Based Learning in a Family Health Nursing Master's Course

**DOI:** 10.1177/23779608251379102

**Published:** 2025-10-27

**Authors:** Catarina Afonso, Odete Amaral

**Affiliations:** 1 Polytechnic Higher Education – School of Health of Santarém IPSantarém. Integrated Researcher at RISE-Health – TL7 – Community Health Prevention, Instituto Politécnico de Santarém, Escola Superior de Saúde de Santarém, membro integrado RISE-Health, Escola Superior de Saúde de Santarém, Instituto Politécnico de Santarém, Santarém, Portugal; 2 School of Health of Polytechnic of Viseu, Portugal. Integrated member of Health Sciences Research Unit: Nursing (UICISA: E), Instituto Politécnico de Viseu Escola Superior de Saúde de Viseu, Viseu, Portugal

**Keywords:** Case-based learning, nursing education, family health, active learning, reflective practice, SWOT analysis

## Abstract

**Introduction:**

Case-based learning (CBL) has gained increasing attention in nursing education for its ability to promote critical thinking, reflective practice, and the integration of theory with real-life clinical scenarios. This article aims to present the exploratory application of CBL in a study carried out during a master's program in Family Health Nursing.

**Methods:**

The methodology included six structured phases, culminating in a strengths, weaknesses, opportunities, threats (SWOT) analysis to evaluate the pedagogical impact.

**Results:**

CBL facilitated nurses engagement, teamwork, and the development of clinical reasoning. Despite initial discomfort with the active learning format, nurses reported significant professional and cognitive growth.

**Conclusion:**

The findings support the relevance of CBL in preparing nursing students for the complexities of family-centered care.

## Introduction

Case-based learning (CBL) is a student-centered teaching method that bridges the gap between theory and practice and promotes critical-reflective thinking and communication skills (Cen et al., 2021). One of the primary advantages of this educational methodology is its ability to highly motivate nurses to solve problems.

Literature states that CBL promotes greater dynamism, interaction, and participation among nurses and support more active engagement, a deeper understanding of the content, and improved knowledge retention (Ali et al., 2018; Gade & Chari, 2013; Nemec et al., 2020; Sebold et al., 2018; Vedi & Dulloo, 2021).

Theoretical teaching remains predominant in many health programs, often leading to a disconnect between conceptual learning and clinical reasoning (Gholami et al., 2021; Sultana et al., 2024). Bridging this gap is a growing priority in the design of nursing curricula, especially in areas like family health nursing, where context-sensitive, evidence-based care is crucial.

In this study, CBL was implemented specifically within a single curricular unit of a Master's program in Family Health Nursing, designed to stimulate nurses’ collaborative engagement.

To support a structured and reflective analysis of the pedagogical strategy, a strengths, weaknesses, opportunities, threats (SWOT) analysis was employed. Widely used in educational research, the SWOT framework facilitates critical evaluation by encouraging learners to articulate internal and external factors influencing learning experiences (Ghazinoory et al., 2011; Hofrichter, 2017). Given this context, the present study explores the implementation and outcomes of a CBL strategy applied within a specific curricular unit of a Master's program in Family Health Nursing. The aim was to explore how the CBL strategy contributed to the development of essential competencies in family-centered care—namely clinical reasoning, communication, collaborative decision-making, and application of family nursing models-among nurses enrolled in a Family Health Nursing program (Figueiredo, 2012).

## Methods

This study applied the CBL methodology to a group of nurses in a Master's program in Family Health Nursing. The intervention was implemented within a single course of a Master's program in Family Health Nursing. It was facilitated by two experienced facilitators, with prior training in active learning methodologies and had participated in workshops on the implementation of CBL strategies. They were also involved in the design and development of the pedagogical strategy.

To assess the impact of this teaching-learning strategy, we employed a SWOT analysis – a structured and systematic (self)evaluation method commonly used in educational and organizational settings. At the end of the course, all nurses were invited to critically reflect on the methodology and share their perspectives regarding its SWOT.

### Interventions

Participation in the SWOT reflection was entirely voluntary and anonymous. The participants were 15 nurses in Family Health Nursing Master. Inclusion criteria included enrollment in the course and attendance of at least 80% of the sessions. No exclusion criteria were applied. Demographic data were not collected, as the analysis focused solely on thematic content from the anonymous SWOT reflections.

This pedagogical strategy was guided by a theoretical framework rooted in family nursing, particularly the Dynamic Model of Family Assessment and Intervention (Figueiredo, 2012) and the principles of family-centered care. It is an essential theoretical and operational framework for systematic assessment and personalized intervention with families (Figueiredo, 2012), providing a reference framework for delivering care in family health nursing, taking into account the stages of the nursing process. These models supported the development of student competencies related to assessing family structures, identifying needs, planning interventions, and evaluating outcomes in a systemic and person-centered way.

### Measures: Process of Case-Based Learning

Group Composition and Instructional Support: Nurses were organized into five groups of 3–4 members, ensuring a balanced distribution of clinical experience and academic performance

The CBL activity was carried out in six stages (Table 1):

**Table 1.** Stages of the Case-Based Learning Activity.

1. Case identification	Nurses select real-life family cases from clinical practice and describe the context.
2. Needs assessment & theoretical framework	Nurses assess the family's health needs, consult clinical documents (e.g., guidelines, articles), and build a theoretical framework.
3. Nursing diagnoses & care plan	Nurses define nursing diagnoses and collaboratively develop care plans.
4. Implementation strategies	Nurses select intervention strategies and discuss expected outcomes.
5. Case presentation	Groups present and discuss their case and care plan in class, encouraging peer interaction and feedback.
6. Evaluation	Nurses critically evaluate the CBL methodology using SWOT analysis.

CBL=case-based learning; SWOT=strengths, weaknesses, opportunities, threats.

Stage 1: Case Identification. Each nurse group identified a real-life family case from their nursing practice experience. They produced a detailed description of the family case and context. This step was designed to meet the course learning objectives, which involve acquiring knowledge related to family health intervention across the life cycle and applying the family nursing theories and models.

Stage 2: Needs Assessment and Theoretical Framework. Nurses identified the family's health needs contributing to the identified problems. They selected key documents (e.g., clinical guidelines, protocols, research articles) to build the theoretical framework around the case. Working autonomously with periodic tutorial guidance, nurses searched for relevant evidence and organized information pertaining to their case.

Stage 3: Nursing Diagnoses and Care Plan Development. Nurses determined the nursing diagnoses for the case and collaboratively developed a comprehensive care plan. A group discussion was then held, during which each group refined their diagnoses and intervention plan through peer and tutor feedback.

Stage 4: Implementation Strategies. Each group selected intervention strategies and discussed expected outcomes relative to the family's needs. This stage was accompanied by further literature review to ensure decisions were evidence based.

Stage 5: Case Presentation. The groups presented and discussed their case and proposed care plan in a classroom seminar. Each presentation (15 min) was followed by questions and discussion, allowing exchange of perspectives among peers.

Stage 6: Evaluation. Finally, nurses engaged in a critical evaluation of the CBL strategy itself. Guiding questions included: To what extent is CBL a positive strategy for teaching and learning? and What challenges does this methodology present for clinical practice? Using SWOT analysis (Hofrichter, 2017), nurses examined the internal strengths and weaknesses of the pedagogical approach and the external opportunities and threats in the learning environment​. The ultimate goal of this evaluative stage was to design improvement strategies to enhance the methodology's effectiveness and, consequently, the quality of teaching in future iterations​.

### Ethical Considerations

This pedagogical study involved the implementation of an active teaching methodology. As such, it did not involve any experimental interventions, clinical procedures, or the collection of sensitive personal data and also no involvement of vulnerable populations, and no risks beyond the normal educational context. In accordance with national regulations and institutional guidelines, this type of educational research is generally considered to pose minimal ethical risk, provided that it does not involve vulnerable populations, invasive procedures, or the processing of sensitive data as defined by the General Data Protection Regulation (European Parliament & Council of the European Union, 2016). No information was collected regarding participants’ health, religion, political opinions, or other protected characteristics. The data used for analysis were limited to nurses’ academic reflections and anonymous feedback on the learning process, which do not allow for identification of individuals and do not infringe on their privacy. Furthermore, informed consent was obtained for the use of anonymized reflections for research and publication purposes, in line with the ethical standards and code of conduct of ethical guidance from educational institutions and in accordance with the General Data Protection Regulation (European Parliament & Council of the European Union, 2016).

### Data Analysis

The corpus of this study was made up of all the written and oral reflections produced by the student groups throughout the CBL process, particularly the outputs from Stage 6 (SWOT analysis). The analysis of this material was primarily qualitative and documentary, based on the content of the case presentations and the SWOT feedback shared in class. During group discussions, nurses debated the information gathered and performed in-depth analysis of their cases, which enabled them to address each clinical challenge more effectively. The case presentations in Stage 5 were observed by both facilitators to note common themes in learning outcomes. At the end of Stage 6, all groups’ SWOT analyses were collected for review.

The SWOT analyses from each group were collected, anonymized, and independently reviewed by the two facilitators, both with advanced training in qualitative research methods. Each facilitator performed thematic coding of the responses according to the four SWOT dimensions. Where discrepancies in interpretation arose, these were resolved through discussion until consensus was achieved. This peer validation ensured consistency and reliability in the analytical process. This structured approach to data analysis enabled a comprehensive understanding of nurses’ experiences with the CBL methodology and informed subsequent pedagogical improvements.

By categorizing nurses’ feedback into these four dimensions, we obtained a comprehensive view of how the CBL initiative functioned and how various factors impacted its success in the teaching-learning process. This information was then synthesized to identify which aspects of the strategy to maintain or strengthen and which aspects needed adjustment in future courses.

## Results

The study involved a total of 15 nurses, aged between 24 and 37 years, with the majority being female (80%). All participants were registered nurses with clinical experience in community or family health. Nurses were organized into five groups, each composed of 3–4 members, with efforts made to balance clinical experience and academic performance within groups.

In developing the care plans, nurses consulted key policy, guidance documents and evidence-based clinical guidelines relevant to family health. Incorporating these resources ensured that the proposed interventions were aligned with current best practices and health system priorities. For example, some groups integrated family nursing interventions like family conferencing, psychoeducation, and positive reinforcement strategies, in line with recommendations from health authorities. After the case discussions, the nurses conducted a critical reflection on the CBL methodology using SWOT analysis.

A synthesis of the findings, supported by selected nurse quotes, is presented below: Strengths: Nurses identified several strengths of the CBL approach: Incremental, phased study that allowed progressive application of learning. Effective integration of theory with practice throughout the case work. Active engagement of nurses, leading to high motivation. Development of clinical skills and critical thinking abilities. Promotion of collaborative teamwork and group problem-solving. Exposure to a diversity of approaches and opinions based on each nurses's professional experiences. “*For the first time, I felt that what I studied in theory actually shaped what I did in practice.”*

Weaknesses: A notable weakness was the initially unfamiliar methodology, which caused some discomfort and required additional time for nurses to adapt. A learning curve was evident as nurses adjusted to a more self-directed, case-driven format. “*In the beginning, it was hard to know where to start – we weren’t used to building everything from scratch.”*

Opportunities: The exercise presented opportunities such as Development of transversal competencies (e.g., communication, leadership, self-directed learning). The promotion of active, continuous, and autonomous learning habits beyond the classroom. Adaptation of the method to different learner profiles and experiences, making learning more personalized and relevant*. “This project pushed me to search, analyze, and apply knowledge — skills I now use in my daily work.”*

Threats: Among external threats, nurses noted: Some lack of preparation or limited awareness of active learning methods among nurses at the outset. Initial resistance to pedagogical change, given many were more accustomed to traditional teaching formats. Constraints like limited time or heavy workloads that could impede full engagement with the case activities. “*The timing was tough. Between shifts and assignments, engaging fully was a challenge.”*

During the reflection, nurses overwhelmingly emphasized the importance of facilitator support and feedback in the CBL process. They noted that the teachers’ guidance and ongoing facilitation were essential for them to recognize and leverage the strengths of the methodology. Facilitator presence helped alleviate discomfort with the new learning approach and kept groups on track, especially during the early stages of case development. *“What made a difference was knowing that the tutors were guiding us. Without them, we would have been lost.”*

## Discussion

In Family Health Nursing, our findings are consistent with a growing evidence base showing that CBL develops the integrated skill set clinicians need for practice—clinical reasoning, problem-solving, and the transfer of knowledge to real cases (Cen et al., 2021; Sultana et al., 2024). In our context, nurses reported greater awareness of family health needs and an enhanced ability to derive appropriate nursing interventions through collaborative analysis, a pattern echoed in qualitative and classroom studies where CBL strengthens care planning and knowledge construction (Burucu & Arslan, 2021; Nemec et al., 2020; Sebold et al., 2018). The process also fostered metacognition and reflection-in-action within a supportive learning environment—nurses continuously monitored and adapted their thinking during case work—aligning with literature that links active, case-driven approaches to gains in metacognitive awareness, self-directed learning, and “soft-skills” essential to clinical practice (Chan et al. 2021; Gade & Chari, 2013; Vedi & Dulloo, 2021). Taken together, these capabilities—situational awareness of family needs, collaborative intervention design, and adaptive expertise—are foundational for advanced practice nurses who must navigate complex family dynamics and community health challenges (Ali et al., 2018; Gholami et al., 2021).

The use of SWOT analysis provided a structured opportunity for nurses to perform a meta-evaluation of their learning process. The SWOT feedback clearly indicated that the CBL strategy had a predominantly positive impact on learning outcomes and skill development. Strengths and opportunities outweighed weaknesses and threats, suggesting that overall, the methodology was beneficial and well-received by nurses.

Notably, the SWOT exercise helped identify specific weaknesses (such as initial discomfort with an unfamiliar methodology) that can be proactively addressed in future implementations. This finding supports previous work by Macedo et al. (2018) and Amaral and Boery (2020), who demonstrated that SWOT can be an effective tool in evaluating teaching methods and guiding pedagogical decision-making. By concisely categorizing feedback, SWOT allowed both nurses and facilitators to pinpoint areas for improvement and to strategically reinforce the strengths of the methodology while mitigating its limitations. Within nursing education, the SWOT framework is increasingly recognized for encouraging critical reflection on teaching-learning experiences and contributing to continuous curriculum improvement (Hofrichter, 2017).

Some educational interventions have also incorporated SWOT or similar reflective tools as part of nurses’ active involvement in evaluating learning methodologies. For instance, Burucu and Arslan (2021) used qualitative methods to explore nursing students’ reflections on the integration of CBL with the nursing process, and nurses were able to offer practical suggestions to improve course design. In our context, nurses proposed orienting sessions early in the semester to clarify expectations and emphasized the importance of sustained faculty mentorship throughout the case development stages. In this way, the SWOT analysis in our study served a dual purpose: it functioned as both a learning activity—enhancing critical reflection—and as a formative tool for teaching evaluation and improvement. This aligns with continuous quality improvement principles in education, where feedback loops are established to constantly adapt and refine pedagogical strategies (Hofrichter, 2017).

## Strengths and Limitations

The positive outcomes observed—including integration of theory and practice, improved critical thinking, and enhanced teamwork—indicate that CBL effectively contributed to the development of key competencies in Family Health Nursing. These findings are consistent with current literature on active learning in nursing education. Recent studies have demonstrated that shifting from traditional lectures to case-based or other active formats leads to significant improvements in nurses’ critical thinking, problem-solving, and communication skills (Chan et al., 2021; Shohani et al., 2023; Sultana et al., 2024).

This study has several limitations that must be acknowledged. First, it was conducted within a single curricular unit in one institution, limiting the generalizability of the findings. Second, the sample size was small and demographically homogenous. Furthermore, although the analysis was qualitative and interpretative, it lacked triangulation with other data sources such as individual interviews or observation records.

To enhance rigor and trustworthiness, we adopted several strategies including dual coding of SWOT data, iterative discussion among facilitators to reach consensus, and transparent documentation of the analysis process. Future studies should incorporate more robust qualitative designs—such as focus groups or longitudinal follow-up—to deepen understanding of learning outcomes. Moreover, transparency in reporting was prioritized through the inclusion of direct nurses quotations and frequency references, which support the interpretation of the qualitative data and reflect the participants’ lived learning experiences.

## Implication for Practice

By combining CBL with reflective tools like SWOT, our study confirms the potential to develop *metacognitive skills*-that is, nurses not only learned *what* to do in clinical situations, but also *how* they learn, and how to improve both their thinking and practice. These capabilities are increasingly considered essential for a resilient, adaptable healthcare workforce.

This pedagogical strategy also aligns with international standards for nursing education, such as the Commission on Collegiate Nursing Education (CCNE, 2018) and the Accreditation Commission for Education in Nursing (ACEN, 2017), which emphasize student-centered learning.

Finally, our results are in line with a broader trend in nursing education towards student-centered and competence-based approaches. Although conducted at a master level, supports this broader pedagogical shift and suggests that CBL is an effective and replicable strategy for training reflective and competent family health nurses.

We recommend that nurse educators consider incorporating CBL into their curricula and continue to evaluate its outcomes. Future research could further explore this methodology across different nursing specialties and educational levels, as well as investigate long-term effects on clinical practice performance. By continuing to innovate and critically appraise our teaching strategies, we can better prepare nurses to meet the evolving challenges of healthcare with expertise, confidence, and a reflective mindset.

## Conclusions

Overall, CBL aligned well with principles of adult learning, providing contextualized and engaging experiences that facilitated the growth of clinical reasoning and problem-solving abilities. This pedagogical strategy encouraged a cyclical process of learning: nurses analyzed and reorganized information, applied it to solve problems and formulate care plans, and then reflected on the effectiveness of their approach. The CBL methodology implemented in this Family Health Nursing master's course proved to be highly beneficial in developing a range of clinical and transversal competencies.

By working through realistic family cases, nurses practised integrating theoretical knowledge with practical decision-making and experienced the importance of critical reflection in clinical practice. The addition of SWOT analysis further reinforced their reflective and evaluative skills, enabling them to identify areas of personal and methodological improvement.

This study highlights the positive impact of CBL nursing nurses in a family health context. The strengths observed—from enhanced engagement and critical thinking to better integration of theory into practice—suggest that CBL, supported by tools like SWOT for reflection, can significantly enrich nursing education.
